# Digital inclusion – invisible work and grey zones for nurses, acting as frontline workers

**DOI:** 10.1186/s12913-026-14040-0

**Published:** 2026-01-20

**Authors:** Carl Erik Moe, Søren Skaarup

**Affiliations:** 1 Department of Information Systems, University of Agder, Universitetsveien 25, Kristiansand, 4630 Norway; 2 IT University of Copenhagen, Rued Langgaardsvej 7, København S, 2300 Denmark

**Keywords:** Digital inclusion, Nurses, Frontline workers, Invisible work, Professional ethos, Nursing ethics

## Abstract

**Background:**

The high degree of digitalization in Scandinavian countries has led to challenges for those who find themselves digitally excluded, facing several barriers to participation in society. Many may receive help from “warm experts”, i.e. family members or friends, but that is not possible for all. Different groups of frontline government workers may also act as helpers, even if they do not have assistance with digital public services as a core part of their remit. In Norway, health personnel often care for citizens in their home settings and may frequently be asked to help with digital challenges.

**Aim:**

This study aims to explore the nature of the work that nurses do in helping citizens with digital challenges, and challenges that they face in doing this.

**Methods:**

This explorative study is based on a survey of 3.044 nurses working in the Norwegian municipal sector. The survey constitutes a combination of a qualitative (open ended questions) and quantitative approach (closed questions), The open-ended questions were thematically analyzed and the close questions were used for descriptive statistics.

**Results:**

The findings show that nurses do considerable invisible work in helping patients with digital technology. They frequently help with both basic hardware and software challenges, as well as with specific services, some of which are related to health. Many do this driven by their professional ethos, believing this is vital to patients´ well-being. This takes time from other (core) tasks. They often worry about the help they provide and find themselves in a grey zone exacerbated by a lack of guidelines and support from peers and colleagues.

**Conclusion:**

The paper contributes to a deeper understanding of the invisible work nurses do due to digitalization of public services, and why they do this. Furthermore, the paper highlights the grey zone which many nurses must navigate, and the challenges they encounter. Although our findings are from a specific context, they may also apply to nurses and healthcare workers in other countries with high degrees of digitalization, and to some extent to other groups of frontline workers. Implications include better digital training but also more concrete guidelines for nurses on what to do when asked for digital help.

**Supplementary Information:**

The online version contains supplementary material available at 10.1186/s12913-026-14040-0.

## Background

Digitalization provides opportunities for more efficient [1], holistic and transparent government [2] with opportunities for businesses reaching customers worldwide [3], and easier access for citizens to public information and services [4]. Scandinavia is at the forefront of digitalizing public services [4, 5] and recognized as leading the way in digitally transforming healthcare [6] for the general population. Digital services are understood as a service executed in full by a technical system [7], and service is understood as “a process by which the provider fulfills a mission for a client so that value is created for each of the two stakeholders” [8]. Digital health has the potential to revolutionize the way healthcare is provided, shifting the balance of power from healthcare professionals to patients and citizens [9]. An overriding vision for governments in Norway and other Scandinavian countries has been “digital first,” with the internet as the main service channel [10], and a similar “push” is evident in large parts of the EU [11]. This has shifted parts of the workload and responsibility from the government to individual citizens, increasing their administrative burden and to some extent making citizens their own caseworkers [12], and has reshaped the roles of frontline workers [13], i.e. the public employees that meet citizens face to face. These structural conditions manifest themselves and must be solved on an individual level, by the citizens and the frontline workers they encounter.

A major challenge facing government digitalization and digital-first strategies, is the digital divide. Many citizens experience challenges with using digital services and channels, and the number of citizens who are digitally excluded is significant in many countries, creating a “digital underclass” [14] who face a number of barriers to their participation in society. The Norwegian digitalisation strategy [15] indicate that as many as 850,000 or 16% of the population are unable to participate digitally or digitally vulnerable. According to Digitaliseringsstyrelsen, as many as 17–22% of the adult Danish population will always be considered “digitally vulnerable” [16]. Other European countries face similar concerns [17, 18]. Our understanding of the digital divide has evolved from early studies focusing on lack of access (see e.g [19]), to somewhat later studies focusing on internet skills and usage [20, 21], and finally to the outcomes of internet use [22–24]. Many factors may contribute to citizens finding it hard to do things online on their own, such as motivation and skills [25], language skills [26], bureaucratic competence [27], domain skills [28] and cognitive resources [29]. Digital divides are often related to fundamental mechanisms of inequality, and the people most at risk of digital exclusion are also the ones most at risk of social exclusion [30]. Previous research shows that elderly face challenges in learning new digital technologies [24], but also that not only elderly are at risk for digital exclusion [31].

We apply the term vulnerable citizens in line with [32] to digitally excluded citizens who do not have access to stable internet or a computer or who are unable to interact with digital technology. Vulnerable citizens frequently require help not only with bureaucratic tasks, but also with technical support [33]. Without help, citizens risk losing welfare services they are entitled to and becoming even more vulnerable [4]. Some may be able to obtain help from family or friends or other “warm experts” in their social networks, but that is not possible for all. Warm experts are informal helpers, typically family or friends, who may be experts to some degree in applying digital technology, and who help due to their family or friendship relationships [34].

Intermediary organizations, such as tax mediators or libraries, may play a role as helpers in some contexts [35], tax mediators can help with tax issues, and librarians can help citizens visiting the library in navigating digital services in general, without getting access to sensitive information. Different groups of frontline government workers may also act as helpers, even if providing such help is not a core part of their remit or professional toolkit.

Tummers et al. [36] draw a picture of conflicting expectations and needs, that frontline workers may have to “cope” with when helping citizens, resulting in stress. We need to understand the challenges providing digital help may pose for these workers, how they arise and how they can be addressed. One such group of frontline workers is nurses and nurse assistants [37], nurses being the focus of attention in our study. Municipal nurses work with some of the most vulnerable groups of citizens and may be the only professionals whom these citizens meet on a regular basis as they often care for patients in their homes or nursery homes. When caring for them, nurses may be asked to help with other tasks, including digital technologies and services. We have not been able to find any literature that studies general digital help provided by nurses.

### Aim

In this study, we aim to understand the nature of the work that municipal nurses do in helping vulnerable citizens with digital challenges and challenges they may encounter in doing this. Understanding this has policy-implications in that it can help determine what types of help are reasonable for nurses to provide, what types of help should be available for citizens from other sources and what skills and resources nurses may need to confidently provide the help they should provide.

### Theoretical concepts

In addition to the drivers of citizens’ need for help, we have chosen two important concepts to help us understand the nature of the digital help nurses provide and what drives their willingness to provide this help, even when it transcends the formal expectations of the job. These are the concepts of “ethics of care” and “invisible work”.

Nurses develop a professional identity through their education and redevelop this through their working life [38]. “Ethics of care” may help explain decision-making related to helping vulnerable citizens with private, non-health related issues. Ethics of care can be understood as what motivates people to help others even when they have no obligation to do so [39]. “Ethics of care” is an important pilar in nursing ethics. Nursing ethics provides an ethical code for nursing practice, outlining what is acceptable nursing conduct [40]. One of the basic principles in nursing ethics is beneficence, which implies doing positive things to help patients, but other ethical principles including autonomy, justice, and confidentiality are also important [41]. Professional ethical codes are an important part of healthcare [42], and The International Councils of nurses has developed a code of ethics for nurses [43].

Changes in the boundaries of practice, such as expanding the scope of the help provided, could lead to changes in training. But, despite the need for nurses’ knowledge and skills in digital technologies, adoption of recommended curriculum changes has been slow and there is a lack of understanding of how digital capability is incorporated into nurses’ workplaces internationally [44] and pedagogical models designed to teach an entire process for the acquisition, measurement, and maintenance of technological literacy are lacking [45]. We anticipate that the majority of practicing nurses have gotten their nursing education many years ago.

Another reaction to unplanned expansions of the scope of work could be local norms and guidelines. Hupe and Van der Krogt [46] specify three sorts of “action prescriptions” that may help constrain the scope and nature of professional work: formal rules, professional norms, and expectations from society – hence guidelines from an employer or common understandings among a group of employees may limit the scope of the help given. Hupe and Buffat [47] specify some action enablers for deciding whether to e.g. set aside time for a task, including training, education, professional experience, time, information and staff, these are included in our sensitizing framework (below, Table 1). Experience may also help balance clients’ needs against institutional frameworks and local guidelines [48].

When nurses help with digital technologies that are not closely related to their core nursing tasks, they perform invisible work. The term invisible work was coined by Daniels [49] to describe those types of women´s unpaid labor that were culturally and economically devalued.

Allen [50] used the term invisible work for the “organizing work” nurses perform to manage their patients’ care. This phenomenon has been studied in different settings and contexts. Studies from hospitals have revealed that the implementation of health information technology may lead to nurses taking over workload from unit clerks and being assigned more documentation work [51], and to an increase in time spent on documentation and decrease in the time nursing staff spend with their patients [52]. A case study of three settings in Denmark [53] revealed that standardizing technology creates invisible work (such as preparation, acknowledging citizens’ efforts, increased documentation) and workarounds (deviating from standard procedures or making excuses for the technology).

A case study from Swedish hospitals showed that eHealth applications may create additional invisible labor for nurses [54], both related to their use of data and data quality, and to helping patients use digital devices. Montañés et al. [55] argued that much of the care provided by nurses is unrecorded, “invisible” and can be seen as an extension of their role as caregivers. Some of the invisible work that Oudshoorn [56] found when interviewing staff involved comforting and reassuring patients about their ability to master new technology. These activities were not covered in their training, or in the implied script of the technology. Kilponen et al. [57] found invisible work in a survey of municipal healthcare employees in Finland and find that this may lead to burnout and have negative effects on work engagement and meaningfulness of work.

In summary, driven by their need for help in navigating the digital world, citizens may turn to municipal nurses visiting them in their homes, and driven by a professional ethos that calls for help beyond their formal role and skill set, nurses may perform invisible work helping citizens with these needs. This may lead to challenges and dilemmas that the nurses will have to navigate.

To understand the nature of the work that municipal nurses do in helping vulnerable citizens with digital challenges and challenges they may encounter we ask the research question: *How much and in what ways do municipal nurses help citizens with their digital challenges and how do the nurses experience this invisible work*?

Doing this includes examining the following:

1. What digital challenges do citizens ask nurses to help with?
2. What do nurses help with and to what extent can this be characterized as “invisible” work?
3. What challenges and dilemmas do this invisible work lead to?
4. What role do professional ethics, skills and experience play in nurses’ willingness and capacity to help?

This paper contributes by exploring the invisible work that municipal nurses do in bridging the digital divides created by the rapidly developing digitalization of society, how this work is driven both by citizens’ needs and the professional ethos of nurses, and the challenges and dilemmas this work creates.

## Methods

Based on the theoretical background and previous research we have developed a sensitizing framework, shown in Table 1 below. Sensitizing in this context implies concepts that offer ways of seeing, organizing, and understanding experiences [58], organized into a tentative framework that can be applied as a guideline for the design of data collection and as a point of departure for data analysis. The table outlines themes that are relevant to investigate to answer the research question.

**Table 1:** Sensitizing theoretical framework guiding the investigation

Dimension		Sources	Themes to investigate
**Citizens’ needs** – what drives the need for help		[4, 6, 27, 30]	Why do citizens say they need help? What do citizens need help with?
**Nurses´ capacity to help** – what drives the nurses to help and what capacities do they have to help	The professional ethos of the nurses, nursing ethics	[13, 37–44]	How do the nurses´ professional identity, and norms and values for what constitutes “good service” (nursing ethics) affect their capacity (or willingness) for helping? Why do nurses help vulnerable citizens with digital challenges?
	Nurses´ skills, training, and experience, and time	[39, 45, 46, 49]	What types of skills are needed and expected from vulnerable citizens? What training is needed to: a) Know whether to help vulnerable citizens with digital issues? b) Help citizens with digital challenges What role does experience play in if and how to provide help? Do nurses have sufficient training, skills and experience to help vulnerable citizens? Do the nurses have time to cater to the needs for digital help?
**Nurses´ invisible work”**		[50–57]	How often do nurses help vulnerable citizens with digital challenges? Do all nurses do this work? Why do some refuse doing this?
**Challenges and dilemmas** related to this invisible work		[25, 33, 53]	What worries and dilemmas arise (for the nurses) and how are they handled?

In order to investigate this, we carried out an exploratory case study [59] into the conditions for assisting vulnerable citizens at the front lines of the digital state. The chosen case – nurses working in Norwegian municipalities – can be considered a critical case [60], as it constitutes a setting where help with digital government services is not an explicit part of the job. Many of the nurses working in municipalities care for patients in their private homes or in nursery homes, hence the patients may get to know them well and may trust them to help with private issues. It is a setting where the patients have to deal with daily work tasks, such as paying invoices, filling in government forms or ordering items or services. To investigate these themes, we carried out a survey among municipal nurses in Norway.

Our aim with using a survey was not to achieve statistical representativeness, but as much variation as possible. Collecting data through a survey gave us the opportunity to collect responses from a wide variety of nurses covering a large variety of citizens’ needs, and from a wide variety of municipalities in relation to size and ways of organizing their services. Norway has 357 municipalities ranging from the smallest with 217 inhabitants, to the median with approximately 5,400 inhabitants and the largest with over 700,000 inhabitants. This variation would have been difficult to capture with interviews.

We developed and applied a survey with 38 questions, combining a qualitative survey approach with a quantitative approach [61]. The survey included 25 closed and 13 open-ended questions. Marsland et al. [62] calls this a “concurrent, enriching design”.

The questions were designed to cover all themes outlined in Table 1, citizens’ needs and expectations, nurses’ capacity to engage, the invisible work they do in helping with digital challenges, and the worries and dilemmas related to doing this invisible work. The closed questions allowing us to gauge the scope and scale of the themes under investigation, consisted mainly of 5-point Likert scale questions, or in 4 instances we used 4-point sale as the option “Always” was considered unrealistic and not included. There were a few questions with fixed response options, such as “What kind of workplace setting do you work at”, most of these had multiple possible answers. The free text open-ended questions provided qualitative data on the participants’ experiences, attitudes, practices and conditions for providing help. The participants made extensive use of the free-text questions. Surveys are valuable tools in qualitative research and can provide both richness and depth when free-text data are analyzed across all questions, even when the individual answers are short [63]. As this study is qualitative, we do not assess the reliability [64] of the results. Our goal is credibility, dependability and transferability, in line with the recommendations from Stenfors et al. [65].

As part of the development of the questionnaire, we conducted pilot tests with three nurses working in municipal healthcare, which resulted in some minor changes. Furthermore, the Norwegian Association for Nurses was given the opportunity to provide input but did not see any need for changes in the questions. The questionnaire was translated from Norwegian to English and is available on request.

The Norwegian Association for Nurses distributed a link to the questionnaire via e-mail in May 2023 to approximately 39.000 of its members registered as working in the municipal sector. This includes nurses in administration and practicing nurses, e.g., in nursery homes, in home care and child and school health nurses. The reason for targeting this group was that we expected that nurses meeting patients in their home settings might be asked about helping with personal digital issues more than nurses working at hospitals. The respondents received an e-mail explaining the goal and the content of the study and a link to more information on the project and were free to answer at their convenience. We collected informed consent to participate from the respondents as part of the questionnaire. No reminder was sent; hence the nurses were only asked once to respond. We received 3.044 completed anonymous responses from nurses who provided their consent. Table 2 below shows the age distribution. The median age of the respondents was 48 years, indicating that the age of the respondents was slightly skewed toward the older population.

**Table 2:** Age of the respondents

Age	*n*	%
18–29	243	7,99%
30–39	599	19,68%
40–49	775	25,47%
50–59	940	30,89%
60+	444	14,59%
Does not indicate age	42	1,38%
**Grand total**	**3043**	**100**,**00%**

We did not ask them about years of experience, gender or ethnicity. Table 3 below shows the proportion of respondents working in the smallest, the medium-sized and the largest municipalities. The response rate was twice as high in the smallest municipalities as the average (less than 5.000 inhabitants), slightly lower than average in the medium-sized municipalities (5–50.000 inhabitants) and nearly 30% below average in the largest municipalities, hence there is a slight bias towards the smaller municipalities.

**Table 3:** Size of the municipalities where the participants worked

No of inhabitants	*n*	%
More than 50.000	1043	34,26%
Between 5.000 and 50.000	1554	51,05%
Fewer than 5.000	447	14,68%
**Grand total**	**3044**	**100**,**00%**

As seen in Table 4 below, most of the nurses that responded work in either home services (31%) or nursery homes (30%), hence we have to a large extent data from the workplaces we have targeted.

**Table 4:** Type of workplace where participants worked

	*n*	%
Homecare nursing	933	30,65%
Nursery home	906	29,76%
Other (unanswered)	660	21,68%
Local health centre	174	5,72%
School health service	174	5,72%
Administration	125	4,11%
Sheltered housing	72	2,37%
**Grand total**	**3044**	**100**,**00%**

As our responses are all from nurses, the citizens they help are mainly elderly individuals who need health and care services due to e.g. cognitive or physical challenges. Smaller groups of respondents also work with immigrants, kindergarten children and schoolchildren and possibly their parents. The text responses were all analyzed by applying NVivo 14 for thematic coding [66, 67]. As the started off with a model, and with questions related to the themes in the model, we coded deductively at the top level (13 codes altogether, including “worries” which answers the item “Challenges and dilemmas”), but at the second level our coding was inductive (e.g. under “Worries” we coded 18 subcodes, these were concatenated into 10, one of them being “no worries”). All the coding was done by the main author. All the responses to the quantitative questions were analyzed and some of them were cross-tabulated, such as the background variables age, size of municipality and job role vs. worries, and access to guidelines.

## Findings

Over half of the participating nurses (61%) worked either in-home services or in nursery homes, but some worked in local health centers for parents with small children or at nursery services at schools. Hence, the majority of the respondents are in close contact with citizens, many of whom visit them in their home settings. In the following sections we present both general findings and findings from individual nurses, to illustrate the overall picture.

## Citizen challenges driving the need for help

The need for help arises when demands posed by digital technologies and services do not match citizens’ skills and resources. 60% of the respondents work in homecare or in nursing homes. The majority of the citizens whom the nurses in the study encounter are elderly people, and many elderly find it challenging to keep up with ever-evolving digital technologies [24]. However, the nurses also reported that other groups of citizens are challenged by digitalization. This includes citizens with limited cognitive ability, psychiatric problems, substance abusers, or citizens with limited language skills or limited knowledge of how the “system” works in Norway.

The circumstances that give rise to the need for help also vary considerably. The most common situation is that when patients have no one else to ask. The qualitative data provides a rich picture of the problems. Many patients do not have relatives who can help them or will not be seeing their relatives for a long period of time. According to one nurse “*the family lives far away, and the home services are in a way the closest family for many*”.

The need for help may be urgent, as is the case when patients need help in logging into a service, updating the TV or charging the cellphone. However, many nurses report that patients’ needs are substantial and truly should have been covered by someone other than them:

Many of the patients are truly ill, including those with cognitive problems. They can no longer fill in forms or order food. They need home services or family to do that. Are there any questions related to next of kin? In my opinion they are not able to/ will not accept that their father/mother is not able to and does not have knowledge concerning the use of the technology

According to another nurse, help is truly needed: “*Especially the ones that do not have relatives (that can help) are vulnerable when meeting new technology. They are afraid of making mistakes. In these cases, support is important for mastering*”.

Our findings show that the most typical problem that leads patients to ask for help is related to basic use of the technology. When asked to expand on this, nurses reported a number of fairly basic technical issues. One nurse reported: “*Helping charging the cell phone is indirect help (with something else …)*” indicating that this makes it possible for the patient to use the phone to call somebody, and this was echoed by many. Using technology for communicating with family and friends is important, but cell phones is an unfamiliar technology for many. They help because “*they have to call their relatives. Or connect to WIFI because the healthiest (and most updated) is going on Facetime with the family*”. Further needs include the use of cellphones for reading and replying to text messages related to health services. Help was also needed with problems related to TVs, PCs, tablets and other devices, as well as with internet access.

Other typical problems that lead patients to ask for help include difficulties in understanding the content of the services they are supposed to use and in finding the information and services they need.

Help with digital government services was the most frequent task nurses were asked to help with, this included logging on to public services, including the web portal Health Norway where citizens can access medical information related to them, such as e.g. prescriptions, scheduled appointments and their core medical record, and where they also can find medical advice. The qualitative data provide a richer picture of tasks patients need help with. One nurse reported that “*Scheduled appointments from the hospital that goes through Health Norway are only noticed by a minority of patients. If the next of kin does not act, the nursing home often misses information regarding e.g. polyclinical appointments. Hence, they miss appointments*.” Further examples include ordering items from the pharmacy, booking appointments with the GP and using the citizen dialog on HealthNorway.

Many nurses talk about patients needing help with payment services at the GP or at the emergency room. They help with *“claiming reimbursement for travel expenses to hospital examinations even though this is really not part of my work*”. Many need help with self-help apps and health technology. Another common need relates to assistive technology, including security alarms, medical dispensers, remote monitoring, electronic keys and even hearing aids. Nurses also help with digital services for communication between patients and employees, services where e.g., patients can access their “plan” and notify the home services if they will have fewer needs than normal due to e.g. traveling.

Many also talk about needs for help in logging on to other public services (especially the Norwegian Welfare Agency, NAV) and filling in forms. Some ask for help with net banking or ordering food or transportation. Many services have been digitalized, and patients need “*help in understanding ticketing systems and schedules for public transportation*”.

Helping with non-nursing related technologies and services may create expectations that nurses will always be able to help. However, even if many people are totally dependent on digital help, they still show patience. As one nurse noted: “*They never demand help; it is rather that they have a strong wish*”.

More than half of the nurses surveyed do not know of any organized help services in the municipality where they work (e.g., through public libraries, other public services or NGOs). Hence, they are not able to direct patients to other helpers.

## Nurses´ capacity to engage – driving and shaping the willingness and ability to help

Nurses’ capacity to engage is related to whether they have the time, training and skills needed to help and to their professional ethos. We know what formal training nurses typically have, so in our survey, we focused on whether they feel they have the necessary skills to help. Furthermore, we wanted to explore why they help if they do, if their professional ethos affects their decision one way or the other.

Most nurses reported that they have the skills necessary to help citizens to a high degree or to some degree (78% in total). In the youngest age groups (younger than 39 years), slightly more nurses reported having the necessary skills. Moreover, slightly more of the youngest nurses felt confident in their knowledge of digital devices and services (23%, compared to 16% for the average).

At the same time one-quarter of the nurses reported that they sometimes declined to help because they did not have the right competences. Some replied that they had the right skills to a high degree or to some degree but nevertheless still declined from time to time because they lacked the right competence.

The qualitative findings tell us more about *why* nurses do help. They help because “*many patients have no-one else (to ask) than us*”, there are no other help channels, and “*the municipality does not have any system for helping*”.

We found that many nurses have a strong sense of ethics. As one nurse put it; “*It is a natural part of helping …*”. Another one told us that “*Digital services is not a nursing task in my opinion, but of course I will help the patient in whatever he or she is struggling with*”. If they have the time, they often help with issues not specifically related to health, and as one nurse told us: “*Often patients ask for help to turn on the TV. I would say that this is far beyond necessary health services, but of course we help if we have time”*. The nurses help even with services that they are not sure if they are allowed to help with, such as logging in to online banking services, health services, payment services for visits to the GP, and the use of other services that require access to digital ID. As one respondent noted, *“Everything affects the mental health, but not everything will be a part of the decision on care assistance”.*

Many told us they “*never refuse to help”*, some add a slight moderation: “*I try to help as much as I can, but sometimes the tasks are too big or complicated*”; “*I don´t refuse, but I try to find others if I am not able to*”; “*I refuse if the recipient has next of kin or power of attorney*”, still indicating that if the recipient has no-one else to ask, they are willing to help.

## Invisible work related to digital help

We wanted to know whether providing digital help is common, if it is acknowledged in any way, and if it is regarded as part of nursing. Close to 75% of the respondents help patients regularly, and quite a few help with digital tasks several times daily. There were significant variations in the frequency of help, depending on the nurse’s age. More than 90% of the youngest respondents (younger than 30 years) help citizens occasionally or often (up to every day), while this is the case for 62% of the oldest respondents (older than 60 years).

Interestingly most of the responding nurses (80%) regard some of the help with digital technologies and services as a natural part of their job, even though this was not a part of their education, and many of them are clear about this not being part of their job description. A few has help with technology as a core part of their job, such as one who told us that: “*I work quite a bit with assistive technology, and nearly all the help I give in that sense will be part of my work*”. However many find that some of the help they provide goes beyond what they consider part of their job such as helping with answering text messages, connecting to a charger, operating the TV, connecting to Wi-Fi, and ordering a taxi or goods from different stores. One nurse explained that “*The basic staffing is such that all “extra” help affects us helpers negatively. For example we don´t get to drink enough water or use the restroom …*”.

Most of the nurses in our sample do not have any formal guidelines related to how and what digital services they should help citizens with. As Table 5 below shows, younger nurses have less access to formal routines and guidelines at their workplace. Many don´t know if they exist. Formal guidelines are less common in the smaller municipalities, but still, only 16% of the respondents in the bigger municipalities are aware of existing guidelines.

**Table 5:** Do you have formal guidelines?

Response / subgroup, total	Age group					Workplace		
Response/Age	18–29	30–39	40–49	50–59	60+	Home services	Nursing home	Total
Yes	9%	10%	12%	15%	16%	16%	10%	13%
No	52%	58%	59%	58%	58%	57%	53%	57%
I don´t know	39%	33%	29%	28%	25%	28%	36%	30%
**Grand total**	**100%**	**100%**	**100%**	**100%**	**100%**	**100%**	**100%**	**100%**

Interestingly, digital help is an issue that the majority of nurses seldom talk with colleagues about, especially younger nurses (under 39) who rarely discuss what and how to help with digital issues at work. Some are “*uncertain on what they are allowed to help with, concerning electronic id*”. Many reply that “*I don´t want to have access to electronic id and bank account, as I may be accused of fraught*”, indicating that they do not know that they are actually not allowed to access someone else’s electronic id. The youngest nurses are considerably less confident about what they are expected to help with than the average responding nurse, but still the majority find that they have insufficient knowledge about what they should and should not help with.

Many miss guidelines or tell us that they “*help, even if we shouldn´t, because it is vital for the patients*” or that they need “*time, and it is not our job to help with these things*”, indicating an ambivalence – helping even if they are not supposed to. However many more say that they lack *competence and time*, without even questioning whether it is their responsibility to help. Many replied that they ask if relatives or next of kin can help, and some refer to other colleagues.

## Challenges and dilemmas

We expected to find dilemmas and worries with doing invisible work, work beyond what is considered a core nursing task, even more so as most nurses have not been trained in helping with digital services and technology, and as guidelines are often missing. A little more than a third of the responding nurses (35%) are worried that providing help with digital technologies and services takes time away from other important tasks, and again, younger nurses are more worried than the older. The differences between the different age groups are large (see Table 6, below). Some told us about this worry in more detail:

“The digital world is a time thief for us who are involved with the residents. We help as we see and understand their situation, but this takes time from other tasks that truly are more important”, and “If a lot of extra time is needed for one user, I worry that the next on the list has to wait longer, and that the last on the list gets an unreasonable late visit”

Nevertheless, many help or ensure that the users receive help:

If I don´t have time to help myself, I make sure they get the help needed from someone else working in the municipality ……. Is it something that is not urgent, I set aside time to help when I do not have anything else scheduled

Many operate in a grey zone where they do things they believe may not be allowed or may even be illegal or may have unintended consequences. In total, 63% of the responding nurses (Table 6 below) who help regularly reported worrying that they may either:

- do something beyond what they are allowed to do, or
- do something wrong, which may have negative effects for the citizens they are helping, or
- obtain too much insight into the citizens’ personal information.

Younger nurses are more worried about these issues than older nurses are. Moreover, younger nurses lack knowledge of what they are allowed to help with. A closer investigation of the responses also show that nurses working in home services worry more than nurses working in other work settings.

The quotes below illustrate that nurses often assist citizens with digital devices and services beyond what they feel confident in doing or are allowed to help with:

I have been asked to help a resident to pay invoices with his bank code. It was hard to know what to do just then

.. it can be uncomfortable having to know the code for the credit card, as it is illegal. However, sometimes we have to help so that they get to pay necessary things such as food and medicine

**Table 6:** What worries do you have in providing this help? Total no of respondents (*n* = 2340, 76,8%)

Worry / subgroup, total	Age group					Workplace setting		Total
	18–29 years	30–39 years	40–49 years	50–59 years	60 + years	Home serv.	Nursing home	
That I get too much insight into personal issues	49%	40%	34%	27%	19%	45%	29%	32%
That I do something wrong, which may harm the one´s I am helping	38%	28%	23%	20%	13%	30%	20%	23%
That I do something beyond what I really am allowed to	49%	37%	29%	27%	16%	43%	28%	29%
I am worried that this takes time from other important tasks	59%	47%	35%	29%	23%	47%	35%	35%

One nurse told us that he/she “*is afraid that the patient will blame me if something is done incorrectly and carries consequences*”. Another one told us:

I am often asked about things related to banking, loans, welfare services and applications, and get access to information that I believe I am not allowed to know / shouldn´t know. (I) try to abstain from helping, but in some situations, we just have to.

Yet another nurse noted that:

it can be uncomfortable knowing the access code for bank services, as it is illegal. However, sometimes we have to help so they get to pay for necessities such as food and medicines.

Despite these dilemmas, many told us that they help even if they know they are not expected to do so, as some of the nurses put it: “*No patient (in the home services) has a legal right to digital help, but we help anyway.*” “*I work with people who suffer from dementia. They live in their homes and need help with most things. My job is to make their daily life good*” (this nurse even included an icon of love in the reply).

Citizens’ expectations and needs for help, and the lack of formal guidelines and of nurses sharing their experiences with colleagues, combined with a lack of action resources such as e.g. education and training, experience, time and professional ethos creates a considerable grey zone, where nurses experience dilemmas and worries in which they navigate when providing help.

**Fig. 1:**
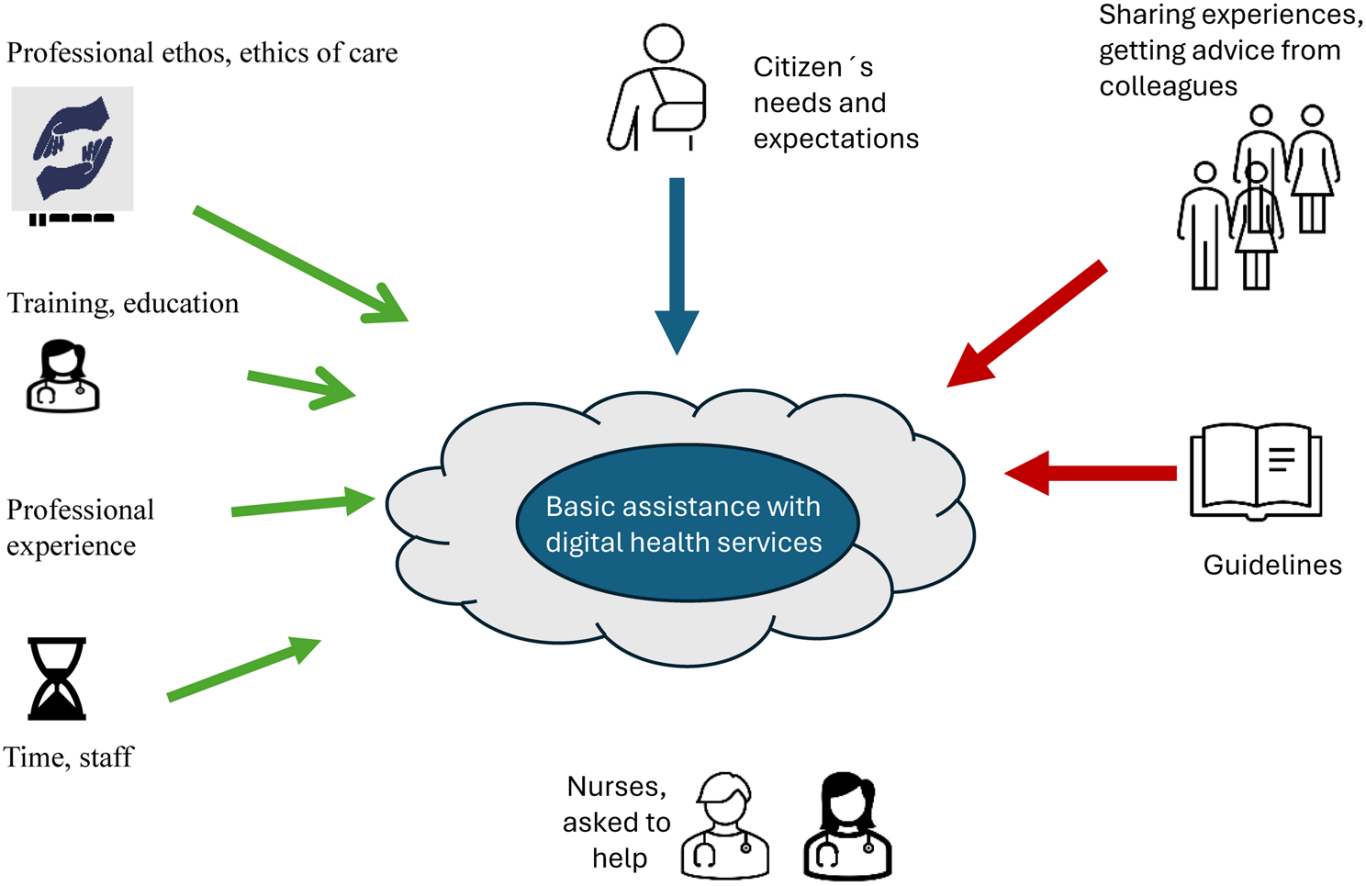
An illustration showing how citizens’ needs and expectations may influence nurses to offer help beyond the basic services, how and to what degree guidelines and support from colleagues may constrain the level of assistance, and how and to what degree action resources such as training, education, professional experience and time (and staff) makes help possible

The illustration in Fig. 1 above shows that citizens’ needs and expectations may influence nurses’ willingness to help beyond the core issues related to digital health services, which may lead them into a grey zone.

## Discussion

Our study shows that nurses working in the municipal sector in Norway play an important role in helping vulnerable citizens with a broad range of digital challenges. This help is not part of their official remit; they have received no formal training for it and typically have no formal guidelines to guide the help they provide. Thus, this study adds “general digital help” as an additional dimension to the invisible work nurses provide. Montanes [55] argue that much nursing work is invisible. However, general digital help is not the invisible work described in the literature, such as work created by the organizing work around the core patient care [50], or administrative work that has shifted from clerical workers to nurses [57]. General digital help is to a large extent work that has been transferred to citizens from both public authorities [12] and the private sector, which is now in part transferred to the nurses, further contributing to the reshaping of the role of frontline workers described by [13].

Our study also shows how this help is driven by two main factors: by the challenges and by needs of the citizens (as documented by several studies such as [22–25, 27]). As shown by [24], these are frequently elderly citizens, who often have no one else to turn to [14]. The other factor driving this help is the ethics of care that are a key part of nursing ethics [38–40]. Thus, the study adds an additional perspective to how ethics of care may drive invisible work.

Finally, the study shows that the combination of significant needs from citizens, strong ethical imperative for the nurses, and lack of formal training and guidelines, creates a grey zone which the nurses have to navigate, a grey zone that contains considerable ethical and legal dilemmas, as nurses when providing help may gain access to information they feel they should not, may do things they are not sure whether its legal and may do things they fear may in the end be detrimental to the citizens.

Navigating this grey zone may lead to stress and conflicting demands for the nurses [36, 55]. Edwards shows that nurses may have considerable training and experience in dealing with grey zones in general [39]. However these are typically grey zones related to the core of their profession. Tummers et al. [36] show how more experienced care managers balance their client´s needs against institutional frameworks and local guidelines. This is in line with the finding from this study that older / more experienced nurses may be better equipped to handle grey zones [44] and more confident in setting boundaries for help, than younger/less experienced nurses. At the same time, the study shows that older nurses may be less technologically confident than their younger colleagues, which lead to the older nurses having a narrower scope for the types of help they provide.

Formal guidelines may help limit the scope of the grey zone, while lack of guidelines may increase it.

In Tummers et al. [36] the care managers have institutional guidelines to help them navigate the grey zone. Hupe and Van der Krogt [46], and Hupe and Buffat [47] also show how formal guidelines, professional norms and professional experience may help reduce grey zones. As this study has shown, the nurses rarely have access to (or know about the existence of) formal guidelines. They also rarely discuss the digital help work with colleagues, something that could allow them to draw on older colleagues with more experience in setting boundaries and contribute to developing shared norms that could guide the help. Huppe & Buffat [47] talk about “action resources” such as training and education that could also assist in delimiting and qualifying the help work. But it appears that there have been little changes in nursing-school curriculums or in training [40, 45], to reflect the need arising from the digital help work. The nurses are to a large extent on their own, left to their own devices.

Thematic analysis and qualitative research are valuable tools also in health research [67], and as a large part of our contributions are based on qualitive results, we apply quality criteria often used for qualitative studies [65] such as variation, credibility and transferability in this study,

Among the total population of 39,000 municipal nurses, 3,044 responded. As we do not know how many nurses actually received the questionnaire, we cannot calculate an exact response rate, but at the lowest end, it would be 7,8%. This constitutes a significant non-response bias. Assuming that workload and relevance are important factors in this selection, there is a risk that we may have underrepresented the busiest nurses and overrepresented nurses who found the subject interesting and important. However, the aim of this study was to understand how the digital help work affected nurses – not its total distribution, and an overrepresentation of nurses who may be more affected may be considered a positive for the study. We did not aim for the study to be statistically representative but to capture as wide a range of experiences from nurses as possible [61]. With more than 3,000 responses, we argue that this has been achieved. The background information on age and workplace does not indicate any significant bias in the response rate. We might expect a higher response rate from nurses who often help citizens with digital technologies and services, as the issues may be more salient to them, but it seems reasonable to assume that non-respondents may experience many of the same challenges even if they are asked less frequently for digital help.

By basing our research question and open-ended questions on our sensitizing framework and on prior theory, and in principle inviting the whole population of municipal nurses to participate in the survey in line with the recommendations from Stenfors et al. [65], we believe we have credible findings.

Surveys are valuable tools in qualitative research [61] and can provide both richness and depth when free-text data is analyzed across all questions. However, ethnographic methods such as interviews, focus groups, and observations may achieve a more in-depth understanding of the experiences of frontline staff and the dilemmas they face. We hope that our work may serve as a point of departure for such further studies.

The context of this study is municipal nurses, primarily working in elder care in Norway. However, we will argue that the needs of the citizens they encounter and the challenges the nurses face in providing help are similar in the other Nordic welfare states. We further expect the same needs and challenges to exists in other areas where vulnerable citizens engage with frontline government workers. What may differ between professions is the degree to which the willingness to help is driven by ethics of care, and the frequency of contact between individual citizens and individual front-line workers. Looking beyond the Nordics, the findings of this study could be relevant where-ever there is a string push for the use of digital services to alle groups of citizens and where the citizens encounter caring-professionals or professionals with a strong ethics of care.

Our approach has several limitations. The initial testing of the survey was limited, which is a challenge in particular to the statistical part of the findings. As we have only descriptive statistics, we would argue that the effects are limited. Although or aim was not representativity but variation, which we have achieved, the low response rate means that there may be considerable variation and blind spots that our approach has not captured. We would however argue that a higher response rate would have added to and not negated our findings.

Our findings contribute to better a understanding of practices, and carry implications for practice, especially for the degree of organizational support needed for the digital help work. The most basic is acknowledging the invisible work. But also important are providing the nurses with some sort of guidelines, and with opportunities for sharing experiences, especially for the younger and less experienced. Better training is also important. Guidelines should be simple – to make them easy to use and remember, and they should be flexible, to allow for the many different contexts where citizens need help. Better possibilities for vulnerable citizens to gain access to services that they are entitled to and better help from the organizations providing these services, may also reduce the need for help.

More research is needed to investigate the role that guidelines and discussions with colleagues play in helping nurses find the right balance between providing citizens with the help they need while staying on reasonably safe and solid ground. In-depth studies should investigate how nurses address the dilemmas and challenges involved. Such studies could also inform the design of guidelines and of various training activities.

## Conclusion

Nurses do a lot of invisible work in helping their patients with digital technology and services. Some of this relates to basic technology, whereas some relates to digital services in general, and some to digital health and welfare services. Many nurses are worried about this help taking time away from core nursing tasks, but many are also worried about the actual tasks they do. This may lead to stress for nurses, it has serious implications for nursing as a profession and may have similar implications for other healthcare employees. Implications include recommending that nurses receive training involving digital health services and digital technology in general and not just limited to medical technologies. As part of this training nurses should be exposed to the challenge of knowing what help belongs to the grey zone, when grey zone help may be advantageous for patients, and when it might not be. Better guidelines are needed, and nurses should discuss these issues in the workplace, especially with younger nurses.

## Supplementary Information

Below is the link to the electronic supplementary material.

Supplementary Material 1

## Data Availability

The datasets generated and/or analyzed during the current study are available from the corresponding author upon reasonable request.

## Abbreviations

EU: European Union
eHealth: Digital health services
Wi-Fi: Wireless Fidelity, wireless network, which gives a user access to the internet
GP: General practitioner, medical doctor
NGO: Nongovernmental organization, usually not a commercial organization
NAV: Norwegian Welfare Association (Arbeids- og velferdsforvaltningen)
