# Supplementary material for: Digital inclusion – invisible work and grey zones for nurses, acting as frontline workers
Source: BMC Health Serv Res. 2026 Jan 20;26:240. doi: 10.1186/s12913-026-14040-0 (PMC12903721; doi:10.1186/s12913-026-14040-0)
Supplement: Supplementary file 1 — Supplementary Material 1 [file 12913_2026_14040_MOESM1_ESM.docx]

| **Question** | **Possible responses** (the open fields are open fields) |
| --- | --- |
| Do you consent to us using your replies in our research | Yes / No. |
| What is your age (Numerical): |  |
| What is your age (Numericals): - Does not want to reply | No. |
| What sort of workplace do you work at? If you have more than one position or work in more than one place, select the place you work at most hours: | Home services / Nursing home / Neighborhood health station / School health service / Administration, allocation of services / Sheltered housing / Hospital / Private health clinic / Other |
| How many inhabitants live in the municipality where your workplace is located? | More than 50 000 inhabitants / Between 5 000 and 50 000 inhabitants / Between 1 000 and 5 0000 inhabitants / Less than 1 000 inhabitants |
| What is your job title? | No predefined alternatives |
| Is there an organized "IT-support" in your municipality (e.g. via the library, Digihjelpen, The voluntary hub, The pensioners´ association, Red Cross or a similar offering)? | Yes / No / I don´t know |
| How often do you have to help your patients and users with digital technologies and digital services? | Several times every day / A few times a day / A few times a week / A few times a month / A few times a year / Never. |
| Approximately how much of the help you give in relation to digital technologies and digital services do you regard as a natural part of your work? | All help is part of my job responsibility / More than half / Half / Less than half / Nothing of the help I give is part of my job. |
| **Feel free to comment on your reply:** |  |
| For how long have you been helping patients/users with digital technologies and digital services in your job? (reply number of years): | 1 / 2-4 / 5-9 / 10-14 / 15-19 / 20+ |
| How often do, in your view patients/users require unreasonable help with digital technologies and digital services? | Always / Often / Sometimes / Seldom / Never |
| What are the typical situations where patients/users need help with digital technologies and digital services? (Put an X in front of everything you find fitting): - Have technical problems | X |
| What are the typical situations where patients/users need help with digital technologies and digital services? (Put an X in front of everything you find fitting): - Are going to do something they haven´t done before | X |
| What are the typical situations where patients/users need help with digital technologies and digital services? (Put an X in front of everything you find fitting): - Are going to do an important task | X |
| What are the typical situations where patients/users need help with digital technologies and digital services? (Put an X in front of everything you find fitting): - Are in an unknown and challenging situation | X |
| What are the typical situations where patients/users need help with digital technologies and digital services? (Put an X in front of everything you find fitting): - Have no-one else that can help | X |
| **Feel free to comment on your reply:** |  |
| What are the typical challenges patients/users encounter that makes them require help? (Put an X in front of everything you find fitting): - Patients/users do not have sufficient technical equipment or access to the Internet | X |
| What are the typical challenges patients/users encounter that makes them require help? (Put an X in front of everything you find fitting): - Patients/users have difficulties in using digital technology | X |
| What are the typical challenges patients/users encounter that makes them require help? (Put an X in front of everything you find fitting): - Patients/users have difficulties in finding the information and services they need | X |
| What are the typical challenges patients/users encounter that makes them require help? (Put an X in front of everything you find fitting): - Patients/users have difficulties in understanding the content of the services they are going to use | X |
| What are the typical challenges patients/users encounter that makes them require help? (Put an X in front of everything you find fitting): - Patients/users are not confident enough to do it on their own | X |
| **Other challenges:** |  |
| What specific tasks do your patients/users normally need help with? (Put an X in front of everything you find fitting): - Logging on to public services, including Health Norway and similar services | X |
| What specific tasks do your patients/users normally need help with? (Put an X in front of everything you find fitting): - Using BankId or something similar | X |
| What specific tasks do your patients/users normally need help with? (Put an X in front of everything you find fitting): - Filling in forms for the public services | X |
| What specific tasks do your patients/users normally need help with? (Put an X in front of everything you find fitting): - Replying to letters from the public services | X |
| What specific tasks do your patients/users normally need help with? (Put an X in front of everything you find fitting): - Ordering goods and services from private entities | X |
| What specific tasks do your patients/users normally need help with? (Put an X in front of everything you find fitting): - Carry out payment or other tasks related to banking and private economy | X |
| What specific tasks do your patients/users normally need help with? (Put an X in front of everything you find fitting): - Using social media | X |
| **Other tasks:** |  |
| How much of the help you offer is done face to face (and not over a distance, such as e.g. telephone or video)? | Almost all / More than half / Half / Less than half / Nearly nothing. |
| Are there situations where you refuse to help patients/users with digital technologies or services? | Yes often / Yes / Sometimes / Yes, but seldom / No |
| What is (are) the typical reason(s) for having to refuse to help? (Put an X in front of everything you find fitting): - The user doesn´t have the necessary digital technology or application | X |
| What is (are) the typical reason(s) for having to refuse to help? (Put an X in front of everything you find fitting): - The user doesn´t have BankId | X |
| What is (are) the typical reason(s) for having to refuse to help? (Put an X in front of everything you find fitting): - The user is to have his/her help from another authority | X |
| What is (are) the typical reason(s) for having to refuse to help? (Put an X in front of everything you find fitting): - The user is to have his/her help from a private authority (e.g. the bank) | X |
| What is (are) the typical reason(s) for having to refuse to help? (Put an X in front of everything you find fitting): - I don´t have the competence needed to help | X |
| What is (are) the typical reason(s) for having to refuse to help? (Put an X in front of everything you find fitting): - It is beyond my job | X |
| What is (are) the typical reason(s) for having to refuse to help? (Put an X in front of everything you find fitting): - I don´t have the time | X |
| **Other reasons:** |  |
| Are there situations where you refuse to help patients/users with some of what they need help for, but not everything? | Always / Often / Sometimes / Seldom / Never |
| What do you do when you have to refuse helping or are not able to help with everything? | I refer to another colleague / I refer to someone else that can help / Something else / Nothing |
| How often do you refer patients/users for help to another public or private entity? | Always / Often / Sometimes / Seldom / Never |
| **Elaborate your reply here. Write what public or private entities you most often refer to (if you do).** |  |
| Is there anything related to what you do when you help patients/users with digital solutions and services that worries you? (Feel free to put more than one X): - That I get too much insight into information on their private matters | X |
| Is there anything related to what you do when you help patients/users with digital solutions and services that worries you? (Feel free to put more than one X): - That I may do something that may have consequences for the person I am helping | X |
| Is there anything related to what you do when you help patients/users with digital solutions and services that worries you? (Feel free to put more than one X): - That I may do something beyond what I am entitled to do | X |
| Is there something related to what you do when you help patients/users with digital solutions and services that worries you? (Feel free to put more than one X): - That it takes time from other important tasks | X |
| Is there something related to what you do when you help patients/users with digital solutions and services that worries you? (Feel free to put more than one X): - No, I don´t have any worries | X |
| Is there something related to what you do when you help patients/users with digital solutions and services that worries you? (Feel free to put more than one X): - Other issues | X |
| **Feel free to elaborate here** |  |
| Does your workplace have any formal rules or guidelines for how to meet the ones who need help with digital technologies and services? | Yes / No / I don´t know. |
| **Feel free to elaborate here** |  |
| Do you discuss how to meet the ones who need help with digital technologies and services at your workplace? | Always / Often / Sometimes / Seldom / Never |
| **Feel free to elaborate here** |  |
| Is there anything of what the patients/users need help with that you find particularly difficult to help with? | Yes / No. |
| **Feel free to elaborate here** |  |
| Do you find you have sufficient knowledge and competence to help patients/users with digital technology and services? | Yes, to a large degree / Yes, to some degree / No, only to a lesser degree / No, not at all. |
| **Feel free to elaborate here** |  |
| What knowledge/skills do you find you lack? (Put an X in front of everything you find fitting): - Knowledge related to technical equipment, technical systems and platforms | X |
| What knowledge/skills do you find you lack? (Put an X in front of everything you find fitting): - Knowledge related to the solutions that patients/users ask for help with | X |
| What knowledge/skills do you find you lack? (Put an X in front of everything you find fitting): - The content of some of the solutions that patients/users ask for help with (the information they offer or rules etc. that apply) | X |
| What knowledge/skills do you find you lack? (Put an X in front of everything you find fitting): - Understanding of how to best help people (pedagogical competence) | X |
| What knowledge/skills do you find you lack? (Put an X in front of everything you find fitting): - Knowledge of what I am allowed to (and not allowed to) help with | X |
| What knowledge/skills do you find you lack? (Put an X in front of everything you find fitting): - Other things | X |
| **Feel free to elaborate here** |  |
| To what degree do you have the necessary tools and resources to help with digital technologies and services? | Completely / To some degree / To too little degree / Not at all. |
| **What tools and resources do you lack when helping?** |  |
